# Reproducibility of peak and time to peak velocity measurements with a high resolution spiral phase velocity mapping (PVM) sequence

**DOI:** 10.1186/1532-429X-15-S1-E42

**Published:** 2013-01-30

**Authors:** Robin Simpson, Jennifer Keegan, David N Firmin

**Affiliations:** 1 NIHR Cardiovascular Biomedical Research Unit, Royal Brompton Hospital, London, UK; 2 Imperial College, London, UK

## Background

Myocardial PVM may be used to assess peak velocities and the times to those peaks (TTP) in order to characterise healthy and pathological regional heart motion [1]. However data on the reproducibility of the technique is limited. A previous breathhold (low resolution) study investigated reproducibility for peak radial and circumferential velocities [2]; however that of TTP measurements was not presented. A higher resolution, navigator-gated study showed that MR had better reproducibility than tissue Doppler imaging for peak and TTP velocity measurements, but only longitudinal velocities were considered [3]. All previous studies have used prospective cardiac gating and late diastolic (atrial systole) peak and TTP velocities have not been investigated. This study presents a comprehensive assessment of the reproducibility of myocardial PVM for assessing peak and TTP velocities throughout the entire cardiac cycle.

## Methods

An efficient navigator-gated spiral trajectory PVM sequence (1.4 x 1.4 mm acquired spatial resolution, 21 ms acquired temporal resolution; nominal acquisition duration 53 heartbeats) was used to scan basal, mid-ventricular and apical short-axis slices in ten healthy volunteers on two separate occasions. Retrospective cardiac gating was implemented, allowing analysis of all major velocity peaks throughout the cardiac cycle, including atrial systole. The reproducibility of peak velocities and their TTP values was assessed by Bland Altman analysis. Analysis of TTP vales was repeated after normalisation to a fixed systolic and diastolic length.

## Results

**Figure 1:**
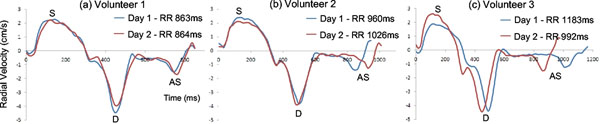
Global radial velocities from the mid slices of three healthy volunteers on two occasions. Volunteer 1 (a) had similar heart rates on both occasions and the two curves match very closely. However volunteers 2 (b) and 3 (c) had different heart rates on the two occasions. TTP systole (S) is not affected much by this change in either volunteer; however the early diastolic (D) TTP is affected in Volunteer 3 but not in Volunteer 2. TTP atrial systole (AS) is affected in both.

Reproducibility data are shown in Table 1. All peak velocity reproducibilities are good, including those for atrial systole which is seen in both radial and longitudinal directions. TTP values expressed as ms from the R-wave show good reproducibility for systolic peaks (-7.1+/-24.1 ms (radial)), but are less reproducible for early (26.3+/-35.7 ms) and late diastolic (25.4+/-88.2 ms) peaks due to heart rate differences on the two scanning occasions (see Figure 1). Diastolic reproducibility can be greatly improved by normalising the TTP values to a fixed systolic and diastolic length (1.3±21.3 and 3.0±10.9 ms for early diastolic and late diastolic peaks respectively), as can be seen in Table 1.

**Table 1:** Mean +/- SD values for all peak velocities and TTP velocities, along with their corresponding interstudy reproducibility (mean +/- signed differences between the two scans)

	Peak velocity (cm/s)	Interstudy reproducibility (cm/s)	TTP (ms)	Interstudy reproducibility (ms)	TTP (normalised) (ms)	Interstudy reproducibility (normalised)(ms)
LONGITUDINAL						

Systolic Peak	5.87±1.89	-0.56±0.87	55.6±10.3	-1.1±6.6	59.9±6.3	-4.5±7.7

Diastolic Peak	-6.50±1.85	0.21±1.54	461.2±39.7	20.6±30.4	483.3±24.1	-3.5±22.2

Atrial-Systolic Peak	-1.66±0.67	0.22±1.08	858.5±137.5	26.6±103.9	869.4±30.6	-1.1±25.6

RADIAL						

Systolic peak	2.38±0.38	-0.01±0.36	108.5±18.0	-7.1±24.1	123.6±16.1	-13.8±27.4

Diastolic peak	-3.61±0.77	0.20±0.56	457.7±68.5	26.3±35.7	487.2±20.2	1.3±21.3

Atrial-systolic peak	-1.59±0.43	0.14±0.42	865.7±129.3	25.4±88.2	875.9±29.9	3.0±10.9

CIRCUMFERENTIAL						

1st systolic peak	-3.52±1.50	0.10±0.50	46.8±12.6	2.4±10.2	48.7±9.8	0.9±9.5

2nd systolic peak	1.40±0.83	-0.20±0.48	135.0±19.5	-2.8±13.6	147.4±21.4	-9.5±7.6

TTP values are shown both as ms from the R-wave (columns 4 and 5) and when normalised to a fixed systolic and diastolic length (columns 6 and 7). The variability and the interstudy reproducibility are both greatly improved for diastolic and atrial-systolic TTPs following normalisation.

## Conclusions

High temporal resolution myocardial PVM with retrospective cardiac gating is a highly reproducible technique for measuring all peak velocities in the cardiac cycle, including those in atrial systole. Absolute and normalised systolic TTP values have comparable reproducibility. However the reproducibility of diastolic TTP values is improved following normalisation to a fixed systolic and diastolic length. This is particularly apparent for the late diastolic peak representing atrial systole.

## Funding

The authors acknowledge the support of Heart Research UK, Imperial College London and NIHR Royal Brompton Cardiovascular Biomedical Research Unit.
